# Response to the association between contact with children and the clinical course of COVID-19

**DOI:** 10.1017/S0950268822001121

**Published:** 2022-07-07

**Authors:** Elizabeth Soyemi, Kenneth Soyemi

**Affiliations:** 1 Illinois Math and Science Academy, Aurora, Illinois, USA; 2 Cermak Health Services at Cook County Juvenile Temporary Detention Center, Chicago, Illinois, USA; 3 Department of Pediatrics, John H. Stroger Jr. Hospital of Cook County, Chicago, Illinois, USA

In ‘the association between contact with children and the clinical course of COVID-19 [1]’, Jannuzzi *et al*. examined the association between contact with children and the clinical course of COVID-19 among COVID-19-positive patients who were hospitalised *vs.* patients who were not hospitalised. The study hypothesis was that non-hospitalised COVID-19 patients have greater contact with children compared with hospitalised patients. The study did not find an association between contact with children and rates of hospitalisation after adjusting for multiple covariates. We also note that the hospitalisation was lower for patients with multiple concurrent child contact types (home and school) when compared with patients with no contact (20 *vs.* 72). Results from this study were surprising as we expected opposite results because of the higher likelihood of infection from multiple sources. We hope authors will provide reasoning to help readers understand the conflicting results.

To understand the role of children in the spread of SARS-CoV-2, it is important to understand the transmission chain (dynamics) of SARS-CoV-2. Most infections in children are asymptomatic, and the number of real-time polymerase chain reaction assay confirmed cases of SARS-CoV-2 infection in children is underestimated because of the high ratio of mild and asymptomatic cases in which testing was not completed [2]. A study by Ustundag *et al*. illustrated that hospitalisation rates were higher in patients without household contact, which is different from Jannuzzi *et al*. findings. One of the reasons for the heterogeneity is that asymptomatic patients with a low-risk contact might not have been tested; some COVID-19 patients ‘without household contact’ were also untested, and the actual number of the patients ‘without household contact’ remained unknown [3].

Transmission dynamics of SARS-CoV-2 infection in children are affected by age groups of household members, exposure intensity, duration of contact, family size and viral load. Paul *et al*. illustrated the greater odds of household transmission by children aged 4–8 years after controlling for testing delays and household size [4]. Soriano *et al*. demonstrated that secondary attack rates were lower in households where children rather than adults had transmitted SARS-CoV-2, and rates were lower during the school period when interactions with other children were expected to increase disease transmission [5]. The age-specific transmissibility of SARS-CoV-2 is a principal factor in disease transmission. There are differences in the viral load of children compared with adults (higher or the same); most children are asymptomatic and may be infectious for a shorter period, making the risk of transmission lower; however, the reduced transmission risk must be balanced by the high number of contacts children have at school and daycare. School contact tracing studies suggest that children and adults are similarly likely to transmit SARS-CoV-2 [6, 7]. Multiple studies in the literature regarding SARS-CoV-2 transmission in children reveal heterogeneity in study conclusions highlighting the role of differential infectivity of paediatric age groups. The heterologous age group infective rates have implications for infection prevention within households, as well as schools/daycare. Public health agencies and health care workers need to understand local household transmission dynamics to mitigate the risk of household secondary transmission prompting the need for further in-depth studies on this topic.
